# Prenatal Diagnosis of Two Common Inborn Errors of Metabolism by Genetic and Mass Spectrometric Analysis of Amniotic Fluid

**DOI:** 10.3389/fped.2022.824399

**Published:** 2022-02-09

**Authors:** Congcong Shi, Sitao Li, Yu Gao, Zhirong Deng, Hu Hao, Xin Xiao

**Affiliations:** ^1^Inborn Errors of Metabolism Laboratory, The Sixth Affiliated Hospital, Sun Yat-sen University, Guangzhou, China; ^2^Department of Pediatrics, The Sixth Affiliated Hospital, Sun Yat-sen University, Guangzhou, China; ^3^Department of Obstetrical, The Sixth Affiliated Hospital, Sun Yat-sen University, Guangzhou, China

**Keywords:** methylmalonic acidaemia, ornithine transcarbamylase deficiency, gas chromatography mass spectrometry, tandem mass spectrometry, prenatal diagnosis

## Abstract

Methylmalonic acidaemia (MMA) and ornithine transcarbamylase deficiency (OTCD) are both intoxication-type inborn errors of metabolism (IEM). Presently, genetic testing is the primary method for prenatally diagnosing these diseases. However, some reports have demonstrated that mass spectrometry approaches can prenatally diagnose some forms of inborn errors of metabolism using amniotic fluid. Therefore, in this study, genetic and mass spectrometry approaches were used for prenatally diagnosing MMA and OTCD. We collected amniotic fluid samples from 19 foetuses referred, 15 cases were referred for MMA and 4 for OTCD. Of the 15 MMA cases, seven were affected, as determined by genetic testing and the metabolite levels; the characteristic metabolites propionylcarnitine (C3), C3/acetylcarnitine (C2) ratio, methylmalonic acid and methylcitrate levels were significantly higher than the reference range. Eight foetuses were unaffected, and the C3, C3/C2 ratio, methylmalonic acid and methylcitrate levels were within the reference range. The C3, C3/C2, methylmalonic acid, and methylcitrate levels in the amniotic fluid significantly differed between the affected and unaffected foetuses (*P* = 0.0014, *P* = 0.0014, *P* = 0.0003, *P* = 0.0014, respectively). Moreover, the homocysteine level increased in the amniotic fluid of affected foetuses with *MMACHC* gene mutations. Of the four OTCD cases, genetic testing confirmed that two foetuses were affected and two were unaffected. However, the characteristic metabolite levels were within the reference range for all foetuses, including citrulline, orotic acid, and uracil. The genetic testing results were confirmed to be correct through the abortion tissue of the foetus and the postnatal follow-up. Our results suggest that mass spectrometry approaches are convenient method for improving the prenatal diagnosis of MMA. The characteristic metabolites C3, C3/C2, methylmalonic acid, and methylcitrate levels in amniotic fluid were reliable biochemical markers for the prenatal diagnosis of MMA.

## Introduction

Methylmalonic acidaemia (MMA) and ornithine transcarbamylase deficiency (OTCD) are both common intoxication-type inborn errors of metabolism (IEM) in China (1, 2). The estimated incidence of MMA is 1.5–3 cases per 100 000 people, and the OTCD incidence is between 1 case per 77 000 people and 1 per 14,000 people (3, 4). MMA is an autosomal recessive organic acidaemia associated with methylmalonyl-CoA mutase and cobalamin metabolic defects. There are five common clinical MMA subtypes, namely mut, cblA, cblB, cblC, and cblD. OTCD is an X-linked inherited metabolic disease caused by pathogenic variants of the OTC gene, and is the most common type of congenital urea cycle disorder (5). MMA and OTCD patients either die shortly after birth or present with acute deterioration, metabolic acidosis, or hyperammonaemia, and later in life, the presentations include intellectual disabilities or abnormal growth and development (1, 6, 7). Early diagnosis and treatment can prevent complications, and prenatal diagnosis is an important way for families with an IEM proband to prevent IEM recurrence.

Presently, tandem mass spectrometry (MS/MS) and gas chromatography-mass spectrometry (GC/MS) are important methods for screening and clinically diagnosing IEM (8, 9). Some reports have demonstrated that MS/MS and GC/MS can prenatally diagnose some forms of IEM using amniotic fluid, such as MMA, propionic acidaemia, isovaleric acidaemia, and β-ketothiolase deficiency (10–14). Therefore, together with gene sequencing, mass spectrometry provides a new method for prenatally diagnosing IEM.

Since the MS/MS and GC/MS methodologies are mature for diagnosing IEMs, this study describes our experiences with prenatally diagnosing IEM by measuring metabolites in the supernatant of amniotic fluid together with direct mutation analysis in MMA and OTC high-risk pregnancies.

## Materials and Methods

### Probands

From January 2019 to June 2021, we recruited 15 unrelated families with MMA probands, 4 unrelated families with OTCD probands, and 73 pregnant women undergoing routine prenatal diagnoses with no history of IEM referrals. Five amniotic fluid samples from the referred MMA cases were collected by the Centre for Prenatal Diagnosis of Xinhua Hospital, Shanghai, China, all other samples were collected by our hospital (The Sixth Affiliated Hospital of Sun Yat-sen University, Guangzhou, China). The probands were diagnosed based on clinical symptoms, GC/MS and MS/MS biochemical results, and genetic testing. Written informed consent was obtained from all participants. The study protocols were approved by the Ethics Committee of the Sixth Affiliated Hospital of Sun Yat-Sen University (ethics committee batch number 2019ZSLYEC-105).

### Amniocyte Samples

The pregnant women with MMA or OTCD probands underwent amniocentesis by an experienced obstetrician; 10 mL of amniotic fluid was obtained from each pregnant woman. The amniotic fluid was centrifuged at 3000 r/min. Then, the amniotic fluid cells were collected for DNA extraction, and the supernatant was collected for mass spectrometry detection in separate sterile test tubes and stored at −80°C until use. All samples were collected before beginning the analyses, and the tests for all samples were performed at the same time.

### Metabolite Detection and Analysis

The characteristic metabolites detected by MS/MS in the peripheral blood of MMA patients were propionylcarnitine (C3), the C3/acetylcarnitine (C2) ratio (C3/C2), methylmalonic acid, and methylcitrate (15). We also assessed the level of homocysteine (Hcy), which is a specific metabolite of the MMA cblC subtype (16); cblC is caused by mutations of the metabolism of cobalamin associated C (*MMACHC*)gene. The characteristic metabolite detected by MS/MS in the peripheral blood of OTCD patients was citrulline, and the metabolites detected by GC/MS were orotic acid and uracil (3, 5).

The diagnostic criteria for characteristic metabolites of MMA and OTCD are consistent between amniotic fluid and peripheral blood. We used the 95th and 5th percentile mass spectrometry results from the 73 cases of normal (i.e. control) amniotic fluid to establish the upper and lower limits of the normal reference interval, the reference range of the different metabolites were displayed in the results section. All samples were sent to the Inborn Errors of Metabolism laboratory of the Sixth Affiliated Hospital of Sun Yat-sen University for MS/MS and GC/MS testing.

### MS/MS Analysis

Several amino acid and carnitine, methylmalonic acid, methylcitrate, and total Hcy determination kits (Zhipu Biotechnology Corporation, Shandong, China) were used for the analysis. MS/MS (Xevo TQ-DIVD, Waters Corporation, Milford, MA, USA) was used to detect the metabolites in 3 μL of amniotic fluid.

### GC/MS Analysis

GC/MS (Q1000, Japan Electronics Co., Ltd.) was used to evaluate orotic acid and uracil in 100 mL of amniotic fluid. The amniotic fluid samples were processed by adding internal standard, deproteinizing, vacuum drying, and trimethylsilyl derivatization (BSTFA/TMCS) before testing.

### Mutation Analysis

The QIAamp DNA Blood Mini Kit (Qiagen Inc., Valencia, CA, USA) was used to extract DNA from the amniotic fluid exfoliated cells, and then, polymerase chain reaction amplification and Sanger sequencing were used to test the candidate variants. The reference gene sequences were obtained from National Centre for Biotechnology Information GenBank database. Short tandem repeat analyses were performed to exclude maternal blood contamination and perform linkage analysis.

### Diagnostic Criteria

Foetuses were considered affected by MMA if the characteristic metabolites in the amniotic fluid were higher than the upper limit of the reference range and the genetic test results of the amniotic fluid cells were consistent with the genetic test results of the proband.

Foetuses were considered affected by OTCD if the concentration of citrulline in the amniotic fluid was lower than the lower limit of the reference range and the concentrations of orotic acid and uracil were higher than the upper limit of the reference range. Further, the genetic test results of the amniotic fluid cells must be consistent with the genetic test results of the proband.

### Statistical Analyses

SPSS version 21.0 (IBM Corp., Armonk, NY, USA) was used for the analyses. The reference range for each analyte was determined by a nonparametric approach, identified the 95^th^ and 5^th^ percentiles from the cumulative levels of the 73 normal foetuses as the upper and lower reference limits, respectively. Wilcoxon rank-sum tests with exact P-values were performed to compare the levels of the characteristic metabolites between affected and unaffected groups. *P* < 0.05 was considered statistically significant.

## Results

### Patient Demographics

We included 19 pregnant women with MMA or OTCD probands aged between 27 and 36 years with gestational ages from 16–21 weeks. We also included 72 pregnant women undergoing routine prenatal diagnosis (i.e. healthy controls) aged between 25 and 38 years with gestational ages from 16–23 weeks.

Table 1 presents the gene mutation spectrum for cases in this study. There were 9 MMA probands with methylmalonyl-CoA mutase (i.e. *MMUT*) mutations, 5 with *MMACHC* mutations, and 1 with metabolism of cobalamin associated B (i.e. *MMAB*) mutations. The OTCD probands all had the ornithine transcarbamylase (*OTC*) gene mutations.

**Table 1:** Gene mutations and clinical diagnosis of 19 probands.

**Family no**.	**Gender**	**Gene**	**Mutation 1 (paternal)**	**Mutation 2 (maternal)**	**Clinical diagnosis**
F1	Female	*MMUT*	c.1741C > T (p.Arg581Ter)	c.385 + 5G > A (splicing)	mut-type MMA
F2	Male	*MMUT*	c.1630_1631del insTA (p.Gly544Ter)	c.753G > T (p.Lys251Asn)	mut-type MMA
F3	Male	*MMUT*	c.1106C > T (p.Arg369His)	c.1159T > G (p.Thr387Pro)	mut-type MMA
F4	Female	*MMUT*	c.424A > G (p.Thr142Ala)	c.323G > A (p.Arg108His)	mut-type MMA
F5	Male	*MMUT*	c.729_730insTT (p.Asp244LeufsTer39)	c.1850T > C (p. Leu617Arg)	mut-type MMA
F6	Male	*MMUT*	c.729_730insTT (p.Asp244LeufsTer39)	c.1663G > A (p.Ala555Thr)	mut-type MMA
F7	Male	*MMUT*	c.323G > A(p.Arg108His)	c.323G > A(p.Arg108His)	mut-type MMA
F8	Female	*MMUT*	c.1280G > A (p.Gly427Asp)	c.1031C > A (p.Ser344Cys)	mut-type MMA
F9	Male	*MMUT*	c.1106G > A (p.Arg369His)	c.1677-1G > A (splicing)	mut-type MMA
F10	Female	*MMAB*	c.289_290delGG (p.Gly97ValfsTer120)	c.566G > A (p.Cys189Tyr)	cblB-type MMA
F11	Female	*MMACHC*	c.482G > A (p.Arg161Gln)	c.80A > G (p.Gln27Arg)	cblC-type MMA
F12	Male	*MMACHC*	c.228_231delTGAC (p.Asp77GlnfsTer22)	c.609G > A (p.Trp203Ter)	cblC-type MMA
F13	Female	*MMACHC*	c.567dupT (p.Ile190TyrfsTer13)	c.609G > A (p.Trp203Ter)	cblC-type MMA
F14	Male	*MMACHC*	c.80A > G (p.Gln27Arg)	c.217C > T (p.Arg73Ter)	cblC-type MMA
F15	Male	*MMACHC*	c.609G > A (p.Trp203Ter)	c.658_660del (p.Lys220del)	cblC-type MMA
F16	Male	*OTC*	-	c.782T > C (p.Ile261Thr)*	OTCD
F17	Male	*OTC*	-	c.867 + 1G > C (splicing)*	OTCD
F18	Male	*OTC*	-	c.103insA (p.Val35SerfsX7)*	OTCD
F19	Male	*OTC*	-	c.512A > G (p.Gln171Arg)*	OTCD

*MMA, Methylmalonic Acidemia; OTCD, Ornithine transcarbamylase deficiency*.

* *The Mutation is Hemizygous*.

### Reference Range

We established a reference range for each analyte using the 95^th^ and 5^th^ percentiles. The following values were considered normal: C3 = 0.16–1.53 μmol/L, C3/C2 = 0.08–0.57 μmol/L, methylmalonic acid 0.97–1.81 μmol/L, and methylcitrate 0.01–0.79 μmol/L. Further, the specific product of cblC also has Hcy 2.40–6.95 μmol/L, citrulline (Cit) > 3.30 μmol/L, orotic acid (Orotate) 0.00 mmol/mol creatinine, and uracil (Uracil) 0.00 mmol/mol creatinine.

### MMA and OTCD Diagnoses

We found no maternal contamination in the amniotic fluid samples by short tandem repeat analysis. Table 2 presents the amniotic fluid metabolite and DNA sequencing results of the 15 pregnancies from MMA families referred. The genetic and metabolite analyses had high consistency. Seven foetuses were affected by MMA, and eight were unaffected. The seven affected foetuses had the same compound heterogeneous mutations as their probands. The C3 (median: 8.47 μmol/L, range: 3.56–15.55), C3/C2 (median: 1.46, range: 0.76–2.20), methylmalonic acid (median: 35.85 μmol/L, range: 9.81–66.78), and methylcitrate (median: 6.48 μmol/L, range: 3.69–17.62) levels in amniotic fluid were higher than the reference ranges. The Hcy concentration in the amniotic fluid of two foetuses with compound heterogeneous *MMACHC* gene mutations was significantly higher than the concentration of the normal range and other affected amniotic fluid samples.

**Table 2:** The characteristic metabolite levels and DNA sequencing results in the amniotic fluid samples of 15 foetuses from MMA families referred.

**Family no**.	**Gestational ages**	**C3** **(μmol/L)**	**C3/C2**	**Methylmalonic acid (μmol/L)**	**Methylcitrate (μmol/L)**	**Hcy** **(μmol/L)**	**Gene**	**Mutation 1** **(Paternal)**	**Mutation 2 (Maternal)**	**Foetus status**
F1	17 weeks	**1.79**	0.25	1.58	0.17	**10.60**	*MMUT*	–	c.385 + 5G > A (splicing)	Unaffected
F2	19 weeks	**8.71**	**1.59**	**35.85**	**4.81**	4.31	*MMUT*	c.1630_1631delinsTA (p.Gly544Ter)	c.753G > T (p.Lys251Asn)	Affected
F3	17 weeks	**13.85**	**1.73**	**66.78**	**7.45**	5.77	*MMUT*	c.1106C > T (p.Arg369His)	c.1159T > G (p.Thr387Pro)	Affected
F4	16 weeks	**7.21**	**1.46**	**15.97**	**6.48**	4.68	*MMUT*	c.424A > G (p.Thr142Ala)	c.323G > A (p.Arg108His)	Affected
F5	17 weeks	**15.55**	**2.20**	**43.04**	**17.62**	6.07	*MMUT*	c.729_730insTT (p.Asp244LeufsTer39)	c.1850T > C (p. Leu617Arg)	Affected
F6	16 weeks	1.09	0.13	1.63	0.02	4.66	*MMUT*	–	–	Unaffected
F7	18 weeks	0.82	0.17	1.15	0.52	2.37	*MMUT*	–	–	Unaffected
F8	16 weeks	1.29	0.18	1.30	0.46	6.29	*MMUT*	–	c.1031C > A (p.Ser344Cys)	Unaffected
F9	18 weeks	**4.94**	**1.01**	**49.96**	**9.90**	6.44	*MMUT*	c.1106G > A (p.Arg369His)	c.1677-1G > A (splicing)	Affected
F10	20 weeks	0.48	0.07	1.24	0.26	4.27	*MMAB*	c.289_290delGG (p.Gly97ValfsTer120)	–	Unaffected
F11	17 weeks	**8.47**	**0.94**	**9.81**	**3.69**	**20.67**	*MMACHC*	c.482G > A (p.Arg161Gln)	c.80A > G (p.Gln27Arg)	Affected
F12	19 weeks	**3.56**	**0.76**	**15.91**	**4.73**	**18.50**	*MMACHC*	c.228_231delTGAC (p.Asp77GlnfsTer22)	c.609G > A (p.Trp203Ter)	Affected
F13	18 weeks	0.77	0.24	1.28	0.10	4.07	*MMACHC*	–	–	Unaffected
F14	18 weeks	0.79	0.14	1.53	0.10	3.84	*MMACHC*	c.80A > G (p.Gln27Arg)	–	Unaffected
F15	17 weeks	0.91	0.14	0.8	0.7	1.3	*MMACHC*	c.609G > A (p.Trp203Ter)	–	Unaffected

*MMA, Methylmalonic Acidemia; C3, Propionylcarnitine; C2, Acetylcarnitine; Hcy, Homocysteine. Reference range: C3 (0.16–1.53) μmol/L, C3/C2 (0.08–0.57), Methylmalonic acid (0.97–1.81)μmol/L, Methylcitrate (0.01–0.79) μmol/L, Hcy (2.40–6.95) μmol/L. Elevated metabolites are shown in bold*.

**Figure 1:**
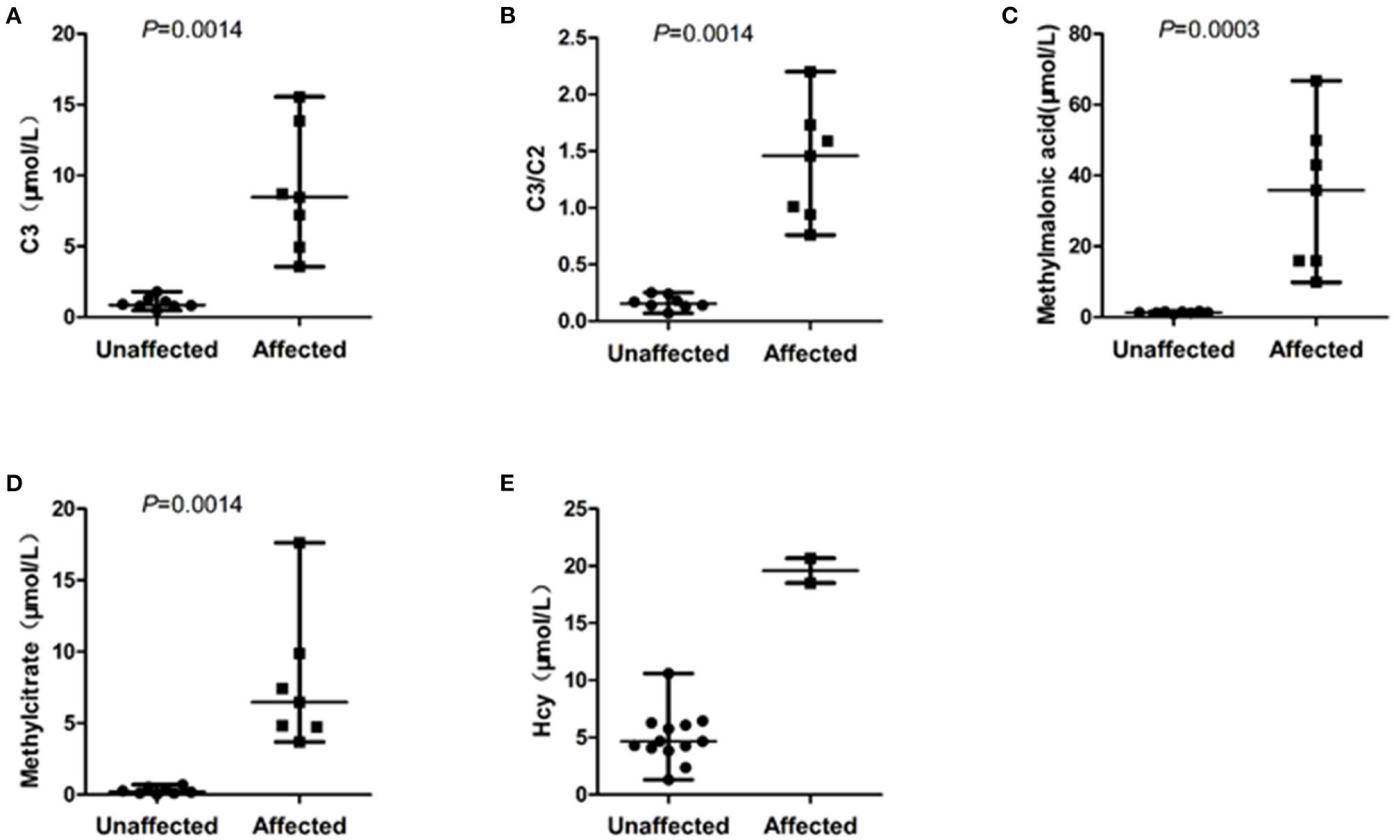
Scatterplot showing the distribution of characteristic metabolite levels in unaffected and affected of 15 cases amniotic fluids referred for MMA. **(A)** The distribution of C3 levels in amniotic fluids between affected and unaffected samples. **(B)** The distribution of C3/C2 ratios in amniotic fluids between affected and unaffected samples. **(C)** The distribution of methylmalonic acid levels in amniotic fluids between affected and unaffected samples. **(D)** The distribution of methylcitrate in amniotic fluids between affected and unaffected samples. **(E)** The distribution of Hcy in amniotic fluids between two foetuses with compound heterogeneous *MMACHC* gene mutations and other amniotic fluid samples unaffected for the *MMACHC* gene. Horizontal lines, median values. *P* values were determined by the Wilcoxon rank sum test.

The eight unaffected foetuses carried one heterogeneous mutation (*n* = 5) or no mutation (*n* = 3), and the median levels of C3, C3/C2, methylmalonic acid, and methylcitrate in the amniotic fluid were 0.87 μmol/L(range: 0.48–1.79), 0.16 (range: 0.07–0.25), 4.17 μmol/L(range: 1.30–10.60), 1.29 μmol/L(range: 0.80–1.63), and 0.22 μmol/L(range: 0.02–0.70), respectively. The C3, C3/C2, methylmalonic acid, and methylcitrate levels in the 7 samples amniotic fluid were within normal ranges. The C3 and Hcy levels in F1 was slightly higher than the normal range, while the levels of the other three metabolites were in the normal range. The C3, C3/C2, methylmalonic acid, and methylcitrate levels did not overlap between the affected and unaffected foetuses (Figure 1).

Table 3 presents the amniotic fluid metabolite and DNA sequencing results of the 4 pregnancies from OTCD families referred. Two foetuses were affected, and two were unaffected based on the *OTC* gene mutations analysis, but the citrulline (8.86–15.27 μmol/L, Reference range: Cit > 3.30 μmol/L), orotic acid, and uracil (0.00 mmol/mol creatinine, Reference range: 0.00 mmol/mol creatinine) levels were in the normal ranges for all samples. The amniotic fluid metabolite and genetic testing results were inconsistent. Thus, the prenatal diagnosis was based on the genetic test results. The abortion tissue of the foetus and the postnatal follow-up confirmed the genetic testing.

**Table 3:** The characteristic metabolites levels and DNA sequencing results in the amniotic fluid samples of four foetuses from OTCD families referred.

**Family no**.	**Gestational ages**	**Cit** **(μmol/L)**	**Orotate** **(mmol/mol creatinine)**	**Uracil** **(mmol/mol creatinine)**	**Gene**	**Mutation**	**Foetus status**
F16	17weeks	15.27	0	0	*OTC*	–	Unaffected
F17	17weeks	8.99	0	0	*OTC*	c.867 + 1G > C (splicing)*	Affected
F18	18weeks	9.86	0	0	*OTC*	–	Unaffected
F19	16weeks	8.86	0	0	*OTC*	c.512A > G (p.Gln171Arg)*	Affected

*OTCD, Ornithine transcarbamylase deficiency; Cit, Citrulline; orotic acid, Orotate; Reference range: citrulline > 3.30 μmol/L, orotic acid (Orotate) = 0.00 mmol/mol creatinine, and Uracil = 0.00 mmol/mol creatinine*.

* *The Mutation is Hemizygous*.

## Discussion

Currently, the standard method for prenatally diagnosing IEM is the detection of genetic mutations in amniotic fluid cells or chorionic tissues, but the disease-causing genes and mutation sites of the proband must be confirmed to complete the prenatal diagnosis. The genetic testing process is also complicated and slow, usually taking 10–25 days. Further, studies have shown that for autosomal recessive diseases, the foetus has only one causative variant as same as the proband, and the genetic testing results of the foetus cannot give a clear conclusion, but mass spectrometry can detect specific metabolite levels in the amniotic fluid, helping the prenatal diagnostic of foetus (13, 17). Importantly, the speed of mass spectrometry is advantageous; the samples in this study were tested by MS/MS and GC/MS within one day. Also, only a small amount of amniotic fluid is required for testing and provides results for multiple metabolites. Therefore, mass spectrometry is a powerful supplement to genetic testing. Current domestic and foreign research indicate that the prenatal diagnosis of genetic metabolic diseases, such as MMA, propionic acidaemia, isovaleric acidaemia, and β-ketothiolase deficiency, can be performed using mass spectrometry (10–14). This study highlights our experience in prenatally diagnosing MMA and OTCD with genetic testing and mass spectrometry.

Some studies have shown that MS/MS detection of C3, methylmalonic acid, and methylcitrate concentrations in amniotic fluid can be used for prenatally diagnosing MMA (10, 12). In this study, the C3, C3/C2, methylmalonic acid, and methylcitrate levels in amniotic fluid were tested by MS/MS in 15 samples from women referred with MMA. These results showed that four biochemical markers significantly differed between the unaffected and affected groups without overlap. Furthermore, all of the three biochemical markers (C3/C2, methylmalonic acid, and methylcitrate) were identical and consistent with the genetic analysis results. However, the C3 levels in F1 was slightly higher than the normal range; the likely reason might be associated with the selection of the reference range. One study suggested that clinical reference ranges may need to be adjusted in response to the degree of overlap between the normal population and the disorder range (18). However, due to the small number of affected samples, it is difficult to establish more reasonable ranges in this manner. Moreover, the normal samples selected in this study are only 73 cases, so the 95th and 5th percentiles were identified as the upper and lower reference limits, respectively. If the number of samples is large enough, the 99.5th and 0.5th percentiles as the upper and lower reference limits may be more reasonable. Nevertheless, our results are also consistent with other related studies, confirming that C3, C3/C2 ratio, methylmalonic acid, and methylcitrate detection in amniotic fluid by MS/MS is feasible for the prenatal diagnosis of MMA.

In this study, we evaluated three MMA subtypes (mut, cblB, and cblC). The cblC subtype, the most common subtype, is caused by biallelic pathogenic variants in the *MMACHC* gene, which encodes a protein involved in the processing and trafficking of intracellular cobalamin. This subtype is also associated with increased Hcy and methylmalonic acid plasma concentrations (17, 19). In our study, five women were referred with MMA type cblC. Three foetuses were unaffected, and two were affected. Except C3, C3/C2, methylmalonic acid, and methylcitrate, the Hcy level in the amniotic fluid of these affected foetuses was significantly higher than the normal reference range and the Hcy level in the other affected MMA subtypes, consistent with the genetic analysis results. This result suggests that the Hcy level in amniotic fluid is an indicator for the prenatal diagnosis of cblC subtype. However, the Hcy levels in F1 was higher than the normal range, the likely reason may be the same as the C3, further data are warranted to prove the reliability of Hcy in the prenatal diagnosis.

OTCD, also known as hyperammonaemia type II, is an X-linked recessive genetic disorder; therefore, almost all hemizygous males develop this disease. Approximately, 20% of female carriers present some neurocognitive disorders due to random X-inactivation. However, the symptoms are substantially milder than those experienced by male patients. Presently, genetic testing is the primary method for prenatal diagnosis of OTCD; although male foetuses can be diagnosed, due to the unpredictability of random X chromosome inactivation, it is impossible to determine whether foetal female carriers have the disease. Prenatal diagnosis of OTCD in female foetuses using mass spectrometry will therefore be difficult. In this study, four women were referred with OTCD, and all foetuses were confirmed to be male through genetic testing. The MS/MS and GC/MS results showed that the citrulline, orotic acid, and uracil levels in all amniotic fluid samples were within the reference range, suggesting that none of the foetuses had OTCD. However, the genetic testing indicated a c.512A > G hemizygous mutation and c.867 + 1G > C hemizygous mutation in the *OTC* gene in two samples, consistent with the proband, there was no mutation in the other two samples. The metabolite and genetic testing results were inconsistent, suggesting that metabolite analyses using amniotic fluid at 16–22 weeks of pregnancy is not suitable for prenatally diagnosing OTCD. This result has not been suggested in other studies, and more data are warranted to prove the conclusion.

In summary, mass spectrometric approaches are a convenient prenatal diagnostic method for pregnancies at risk for MMA. Characteristic metabolite (C3, C3/C2, methylmalonic acid, and methylcitrate) levels in the amniotic fluid were reliable biochemical markers for prenatally diagnosing MMA. A combined genetic and biochemical approach would provide increased diagnostic accuracy.

## Ethics Statement

The studies involving human participants were reviewed and approved by the Ethics Committee of the Sixth Affiliated Hospital of Sun Yat-sen University (Ethics Committee Batch no. 2019ZSLYEC-105). The patients/participants provided their written informed consent to participate in this study.

## Author Contributions

XX conceived and designed the study. CS wrote the manuscript draft. SL, ZD, HH, and XX enrolled the patients. YG performed amniocentesis. CS and SL performed the metabolite test, mutation test, and data analysis. All authors read and approved this manuscript.

## Funding

This work was supported by Guangzhou Science and Technology Plan Project (No. 201604020154) and Guangdong Science and Technology Plan Project (No. 2020A1414010111).

## Conflict of Interest

The authors declare that the research was conducted in the absence of any commercial or financial relationships that could be construed as a potential conflict of interest.

## Publisher's Note

All claims expressed in this article are solely those of the authors and do not necessarily represent those of their affiliated organizations, or those of the publisher, the editors and the reviewers. Any product that may be evaluated in this article, or claim that may be made by its manufacturer, is not guaranteed or endorsed by the publisher.
